# P-236. Impact of a New Dressing Protocol for the Prevention of Central Line-Associated Bloodstream Infections in one Neonatal Intensive Care Unit (NICU)

**DOI:** 10.1093/ofid/ofae631.440

**Published:** 2025-01-29

**Authors:** Katarina Kowatsch, Audrey Larone Juneau, Justine Giroux, Marianne Lapointe, Alina Vlad, Laura Ocampo, Nathalie Audy, Sophie Gravel, Christian Lachance, David Buckeridge, Caroline Quach

**Affiliations:** Department of Epidemiology and Biostatistics, School of Population and Global Health, McGill University, Montréal, Canada., Montreal, Quebec, Canada; Division of Neonatology, CHU Sainte-Justine, Montreal, Quebec, Canada, Montreal, Quebec, Canada; Division of Neonatology, CHU Sainte-Justine, Montreal, Quebec, Canada, Montreal, Quebec, Canada; Division of Neonatology, CHU Sainte-Justine, Montreal, Quebec, Canada, Montreal, Quebec, Canada; Division of Infection Prevention and Control, Clinical Department of Laboratory Medicine, CHU Sainte-Justine, Montreal, Quebec, Canada, Montreal, Quebec, Canada; Division of Infection Prevention and Control, Clinical Department of Laboratory Medicine, CHU Sainte-Justine, Montreal, Quebec, Canada, Montreal, Quebec, Canada; Division of Infection Prevention and Control, Clinical Department of Laboratory Medicine, CHU Sainte-Justine, Montreal, Quebec, Canada, Montreal, Quebec, Canada; Division of Neonatology, CHU Sainte-Justine, Montreal, Quebec, Canada, Montreal, Quebec, Canada; Division of Neonatology, CHU Sainte-Justine, Montreal, Quebec, Canada, Montreal, Quebec, Canada; Department of Epidemiology and Biostatistics, School of Population and Global Health, McGill University, Montréal, Canada., Montreal, Quebec, Canada; Division of Infection Prevention and Control, Clinical Department of Laboratory Medicine, CHU Sainte-Justine, Montreal, Quebec, Canada and Departments of Microbiology, Infectious Diseases and Immunology and Pediatrics, University of Montréal, Montréal, Québec, Canada., Montreal, Quebec, Canada

## Abstract

**Background:**

Newborns in the neonatal intensive care unit (NICU) are vulnerable to infections due to their extremely fragile skin, underdeveloped immune systems, and usual long-term hospitalizations. These infants often require prolonged use of central venous catheters (CVCs). Bloodstream infections can arise from these CVCs and are a major cause of death in the NICU. Every CVC dressing change can damage their fragile skin, increasing infection risk, and potentially resulting in central line-associated bloodstream infections (CLABSIs). Centers for Disease Control and Prevention recommends dressing changes every seven days, more often if visibly damaged or dirty. Our objective was to evaluate the effectiveness of a new protocol and dressing optimized for long-term skin adhesion implemented in the Centre Hospitalier Universitaire Sainte-Justine (CHUSJ) NICU.

**Methods:**

The CHUSJ NICU is a level III-IV NICU with 900 admissions a year from both inborn and outborn infants. CLABSI surveillance is done prospectively by the Infection Prevention and Control team. All CVCs installed in NICU patients are recorded daily. Inclusion criteria were all recorded NICU CVCs from January 1, 2017, to December 31, 2023. Segmented interrupted time-series analysis was conducted according to the three-stage intervention rollout (first for gestational age of 28 weeks or more, then large premature infants, then the entire unit), with a pre-intervention period of January 1, 2017, to May 1, 2021, modeling CLABSIs/1000 CVC days/month and adjusting for mean birth weight.

**Results:**

2653 CVCs and 71 CLABSIs were reported during the surveillance period. Pre-intervention rates were 1.85 CLABSIs/1000 CVC days. CLABSI rate ratios were: during initial intervention rollout 0.448 (95% confidence interval 0.407, 0.492), during the second stage 0.952 (0.932, 0.973), and since complete implementation 0.610 (0.581, 0.639) that of pre-intervention period rates, for the entire NICU population.

**Conclusion:**

With reports of CLABSI rate rebounds during the COVID-19 pandemic and increasing NICU outbreaks from multidrug-resistant organisms with associated high mortality, new infection prevention protocols are crucial to combat these trends in CLABSI rates and severity of case outcomes, especially for the vulnerable NICU population.

**Disclosures:**

**All Authors**: No reported disclosures

